# Weak Negative and Positive Selection and the Drift Load at Splice Sites

**DOI:** 10.1093/gbe/evu100

**Published:** 2014-05-14

**Authors:** Stepan V. Denisov, Georgii A. Bazykin, Roman Sutormin, Alexander V. Favorov, Andrey A. Mironov, Mikhail S. Gelfand, Alexey S. Kondrashov

**Affiliations:** ^1^A.A. Kharkevich Insitute for Information Transmission Problems RAS, Moscow, Russia; ^2^Faculty of Bioengineering and Bioinformatics, M.V. Lomonosov Moscow State University, Moscow, Russia; ^3^Division of Oncology Biostatistics, The Sidney Kimmel Comprehensive Cancer Center at Johns Hopkins, Baltimore, MD; ^4^Laboratory of System Biology and Computational Genetics, Department of Computational System Biology, N.I. Vavilov Institute of General Genetics, Moscow, Russia; ^5^Laboratory of Bioinformatics, State Research Institute of Genetics and Selection of Industrial Microorganism (GosNIIGenetika), Moscow, Russia; ^6^Life Sciences Institute and Department of Ecology and Evolutionary Biology, University of Michigan

**Keywords:** splicing, splice sites, nearly neutral evolution, positive selection, negative selection, drift load

## Abstract

Splice sites (SSs) are short sequences that are crucial for proper mRNA splicing in eukaryotic cells, and therefore can be expected to be shaped by strong selection. Nevertheless, in mammals and in other intron-rich organisms, many of the SSs often involve nonconsensus (Nc), rather than consensus (Cn), nucleotides, and beyond the two critical nucleotides, the SSs are not perfectly conserved between species. Here, we compare the SS sequences between primates, and between *Drosophila* fruit flies, to reveal the pattern of selection acting at SSs. Cn-to-Nc substitutions are less frequent, and Nc-to-Cn substitutions are more frequent, than neutrally expected, indicating, respectively, negative and positive selection. This selection is relatively weak (1 < |4*N*_e_*s*| < 4), and has a similar efficiency in primates and in *Drosophila.* Within some nucleotide positions, the positive selection in favor of Nc-to-Cn substitutions is weaker than the negative selection maintaining already established Cn nucleotides; this difference is due to site-specific negative selection favoring current Nc nucleotides. In general, however, the strength of negative selection protecting the Cn alleles is similar in magnitude to the strength of positive selection favoring replacement of Nc alleles, as expected under the simple nearly neutral turnover. In summary, although a fraction of the Nc nucleotides within SSs is maintained by selection, the abundance of deleterious nucleotides in this class suggests a substantial genome-wide drift load.

## Introduction

When all four nucleotides (alleles) that can occupy a nucleotide site (locus) confer exactly the same fitness, selection, by definition, does not operate at this locus. By contrast, when one allele confers a much higher fitness that all others, this allele will likely be fixed in the population, and strong negative selection will prevent the spread of alternative alleles. However, an intermediate situation is also possible, when allele fitnesses are not identical, but their differences are minor, of the order of approximately 1/*N*_e_, where *N*_e_ is the effective population size. Such a weak selection has little power to prevent the fixation of even deleterious alleles. Then, as long as the per locus mutation rate µ is not too high (µ << 1/*N*_e_), the locus experiences a random succession of fixations of alternative slightly deleterious and slightly advantageous alleles, even when the fitness differences associated with these alleles remain invariant (Kimura and Ohta 1971; Ohta 1992). When the best allele is fixed, we are dealing with weak negative selection in its favor, and when an inferior allele is fixed, we are dealing with weak positive selection that favors the currently absent superior allele (Li 1987; Bulmer 1991; Kondrashov 1995; Charlesworth and Eyre-Walker 2007).

Thus, in addition to the conventional Darwinian situation when positive selection is typically triggered by changes in the fitness landscape, weak positive selection can also appear due to nearly neutral (Ohta 1992) evolution at a locus even if the fitness landscape is constant. Data on fitness effects of mutations suggest that a substantial fraction of them fall into the nearly neutral class (Yampolsky et al. 2005; Eyre-Walker et al. 2006), and a number of considerations suggest that nearly neutral evolution is a frequent occurrence (reviewed in Akashi et al. 2012). Weak positive selection favoring slightly advantageous mutations has been detected in chameleons, *Anolis* lizards, *Monarcha* (bird), *Coraria* (plant), and *Eleutherodactylus* (frog) from an increase in the rate of evolution in a population which recently underwent a substantial expansion (Charlesworth and Eyre-Walker 2007). In general, however, such weak positive selection is hard to observe, because it is usually masked by negative selection, and its overall prevalence remains unclear.

A steady-state turnover of fixations of slightly deleterious and slightly advantageous alleles in a population of a constant size can be detected through patterns in evolution of a large ensemble of loci that all experience approximately the same constant fitness landscape. If, for example, allele A is optimal at all these loci, and allele G is inferior, we will see that A→G replacements are rarer, and G→A replacements are more common, than what is expected under neutrality. From such an observation, we can infer the strength of negative selection against A→G mutations and of positive selection in favor of G→A mutations.

**Fig. 1.—:**
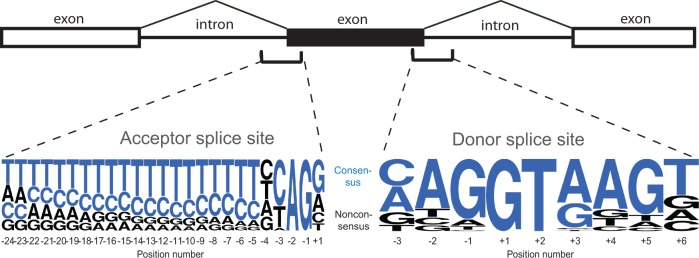
Cn and Nc nucleotides within SSs of *Homo sapiens.* For an intron to be spliced, it requires a pair of functional SSs that involve several nucleotides at the exon–intron boundaries. Both the donor and the acceptor SSs have intronic and exonic parts. The intronic parts (positions +1 … +6 of the donor SSs, and positions −24 … −1 of the acceptor SSs) are longer than the exonic parts (positions −3 … −1 of the donor SSs and position +1 of the acceptor SS). The height of the letters is proportional to the frequency of the corresponding nucleotide at this position. For each SS position, one or two frequent nucleotides were defined as Cn (blue), and the remaining nucleotides as Nc (black). The nucleotide frequencies in *Drosophila melanogaster* are very similar, and the same Cn nucleotides were defined. Sequence logos were constructed using WebLogo (Crooks et al. 2004).

Such ensembles of loci (nucleotide sites) are likely provided by corresponding positions within splice sites (SSs) at exon–intron boundaries. The primary function shaping the sequence of an SS is spliceosomal recognition. Both high level of interspecies conservation (Sorek and Ast 2003; Schwartz et al. 2008; Irimia et al. 2009) and reduced level of genetic variation within population imply selective pressure acting on these sequences (Lomelin et al. 2010). Except some minor classes of SSs (U12-sites, U2-type GC–AG and a few others) (Sheth et al. 2006), most of the SSs are recognized by the same U2-type GT–AG spliceosome, and both the donor and the acceptor SSs are always close to their respective consensuses (fig. 1). We used SSs of constitutive exons to avoid possible conflicting selective pressures connected to alternative splicing regulation (Cooper and Mattox 1997). Thus, it is reasonable to assume that loci at corresponding positions within SSs of different introns all experience similar fitness landscapes, with the consensus (Cn) nucleotides being favored, and nonconsensus (Nc) disfavored, by selection.

## Materials and Methods

We studied the evolution of donor and acceptor SSs of constitutive exons in the human (*Homo sapiens*) and *Drosophila melanogaster* lineages since their divergence from the macaque (*Macaca mulatta*) and *D. simulans*, respectively.

The ancestral states were reconstructed using the maximum parsimony approach, using marmoset (*Callithrix jacchus*) and *D. yakuba* as outgroup species. As the phylogenetic distances between all these species are rather low, multiple hits are rare, and parsimony method is expected to work well. Maximum likelihood inference was done with baseml program of PAML package (Yang 2007; model UNRERST).

A total of 255,002 human acceptor SSs, 261,400 human donor SSs, 46,498 *D. melanogaster* acceptor SSs, and 48,041 *D. melanogaster* donor SSs were extracted from GENCODE release 7 (http://hgdownload.cse.ucsc.edu/goldenPath/hg19/encodeDCC/wgEncodeGencodeV7/, last accessed June 14, 2014) database. After exclusion of the SSs not carrying the canonical dinucleotides (AG and GT for donor and acceptor SSs, respectively) in either the reference, sister, or outgroup species, and of the alternative exons, we were left with a sample of 159,280 human constitutive acceptor SSs, 160,234 human constitutive donor SSs, 40,859 *D. melanogaster* constitutive acceptor SSs, and 41,051 *D. melanogaster* constitutive donor SSs. Each of these samples was subdivided into several subsamples depending on the position within the transcript (ORF-interrupting introns vs. introns within noncoding regions, i.e., UTRs or noncoding RNAs) and strength of the SS (weak, middle, or strong, depending on the fraction of Cn nucleotides; the thresholds were chosen so that an approximately equal number of SSs falls into each category). A total of 39,758 (1,376) cassette-exon donor SSs and 39,644 (1,353) cassette-exon acceptor SSs with canonical dinucleotides were obtained from human (resp. *Drosophila*) genomes, and analyzed separately. We used local recombination rate from Comeron et al. (2012) for *D. melanogaster*, and sex-averaged recombination rate (Kong et al. 2002) downloaded from UCSC Genome Bioinformatics Site (http://hgdownload.soe.ucsc.edu/goldenPath/hg19/database/recombRate.txt.gz, last accessed June 14, 2014) for *H. sapiens*. Gene expression data for *H. sapiens* were obtained and processed as described in (Kurmangaliyev et al. 2013).

For a pair of nucleotides *Z* and *X*, we denote the number of nucleotide substitutions from the ancestral nucleotide *Z* to the descendant nucleotide *X* as *#(Z*→X); the number of cases when nucleotide *Z* was conserved was denoted as *#(Z*→Z).

In each SS position, one or two most frequent nucleotides were defined as Cn, and the remaining nucleotides as Nc (see fig. 1). We calculated the frequencies of substitutions between the Cn and Nc nucleotides *q*(Cn→Nc) and *q*(Nc→Cn) as

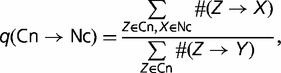


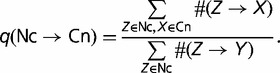

Expected frequencies of substitutions were calculated using the same formulas, but in place of each particular Cn or Nc nucleotide within the SSs, the same nucleotide within a presumably neutral region was considered. We used different such regions for different SS positions: Sequences inside the adjacent intron were used for the intronic part of the SSs, and the third positions of 4-fold degenerate codons within corresponding exon were used for exonic positions of SSs. To use only neutrally evolving positions for our neutral control, we filtered out about one-half of the positions with the absolute value of phyloP score < 0.6 (Pollard et al. 2010) in the human genome. We did not apply an analogous procedure to *D. melanogaster*, because there is no available phyloP score data for insects. However, when neutral controls were constructed from short introns of the *D. melanogaster* genome, which is the fraction of the genome experiencing the weakest selective constraint (Parsch et al. 2010), the obtained results were similar (data not shown).

The 95% confidence intervals for frequencies of substitution were calculated from the binomial distributions using the Clopper–Pearson method (MATLAB function binofit). The 95% confidence intervals for 4*N*_e_*s* were calculated analogously, assuming that the errors in the neutral controls are negligible. The 95% confidence intervals for multispecies conservation *c* were estimated by bootstrapping alignment columns with replacement in a total of 1,000 bootstrap trials. Calculation of the 95% confidence intervals for multispecies conservation was performed as described in Efron and Tibshirani (1993).

The multispecies conservation was defined as follows. For a given nucleotide (or set of nucleotides at a given nucleotide position), we measure the phylogenetic distance *L* (in Ks units) between the reference species *r*, that is, either *H. sapiens* or *D. melanogaster,* and the most phylogenetically distant species *x* such that this nucleotide (or any nucleotide of this set) is observed in all species *i* such that *L*(*r, i*)* ≤ L*(*r, x*)*.* We then average the values of *L* for all corresponding nucleotide positions across all SSs of a given type. For a nucleotide (or a set of nucleotides) at a given SS position, we can then compare its average multispecies conservation (*obs*) with the average conservation of the same nucleotide (or set of nucleotides) within a nearly neutrally evolving region (*exp*) by estimating *c* = (*obs − exp*)/*exp.* Evolutionary distances between species of vertebrates and insects (in Ks units) were calculated from phylogenetic trees taken from UCSC Genome Bioinformatics Site.

Sequence logos in figure 1 were constructed using WebLogo (http://weblogo.berkeley.edu/, last accessed June 14, 2014; Crooks et al. 2004).

## Results and Discussion

### Patterns of Selection on Nucleotides in SSs

**Fig. 2.—:**
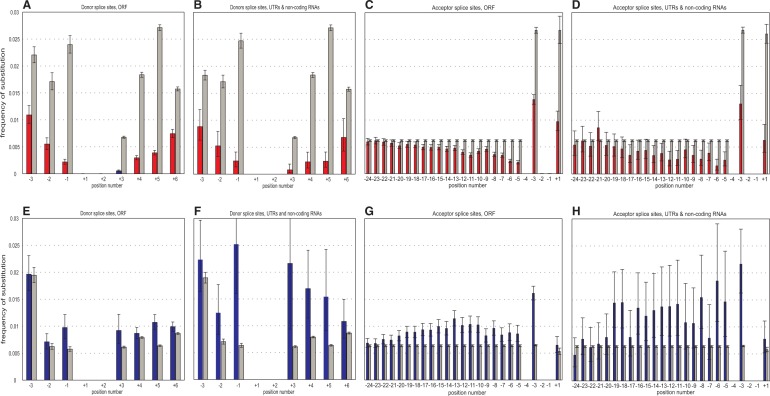
Frequencies of nucleotide substitutions at the *Homo sapiens* lineage since its divergence from the *Macaca mulatta* lineage within different positions of SSs. Horizontal axis, position within the SS relative to the invariant AG (GT) dinucleotide. (*A–D*) Cn-to-Nc substitutions; red, observed; gray, expected. (*E–H*) Nc-to-Cn substitutions; blue, observed; gray, expected. (*A*), (*B*), (*E*), (*F*): Donor SSs; (*C*), (*D*), (*G*), (*H*): Acceptor SSs. (*A*), (*C*), (*E*), (*G*): Coding SSs; (*B*), (*D*), (*F*), (*H*): Noncoding SSs. Error bars are 95% confidence intervals.

**Fig. 3.—:**
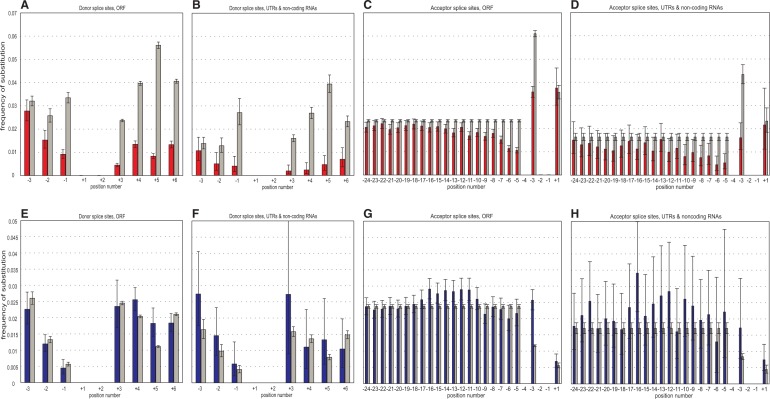
Frequencies of nucleotide substitutions at the *Drosophila melanogaster* lineage since its divergence from the *D. simulans* lineage within different positions of SSs. The notation is as in figure 2.

Figures 2 and 3 present the data on the frequencies of substitutions from Cn to Nc alleles (nucleotides) and vice versa at different loci within SSs, compared with the frequencies of identical nucleotide substitutions within nearby, presumably neutral, control sequences, in the *H. sapiens* lineage since its divergence from the *M. mulatta* lineage, and in the *D. melanogaster* lineage since its divergence from the *D. simulans* lineage. The frequency of substitutions was not defined for positions +1 and +2 of the donor SSs and for positions −1 and −2 of the acceptor SSs, because they are occupied by the invariant canonical GT (AG) dinucleotides; and also for position −4 of the acceptor SSs, where no Cn can be established. We consider separately the “noncoding” SSs within introns that interrupt noncoding RNA segments (UTRs of mRNAs and noncoding RNAs) and the “coding” SSs within ORF-interrupting introns, because they display substantially different patterns.

At all types of loci, Cn alleles are replaced with Nc alleles in the human or the *D. melanogaster* lineage slower than in the corresponding controls (Fisher’s exact test, *P* < 10^−18^; supplementary table S2, Supplementary Material online). At most types of loci, Nc alleles are replaced with Cn alleles faster than in the controls (*P* < 10^−3^ for all samples except *D. melanogaster* donor SSs, where the difference is insignificant; supplementary table S2, Supplementary Material online, and figs. 2 and 3). This indicates that the fixed Cn alleles are protected by negative selection, and the initially absent Cn alleles are favored by positive selection, as expected. At acceptor SSs, the signals of both negative and positive selection gradually disappear around position −22 in the human genome (−17 in the *D. melanogaster* genome), signifying the end of the polypyrimidine tract.

### Strength of Positive and Negative Selection along the SSs

Under weak selection, the probability of fixation of a unique allele with selection coefficient *s* is *u*(*s*) = (1/2*N*)4*N*_e_*s*/[1 − exp(−4*N*_e_*s*)], where *N* is the census population size (Kimura 1983). Thus, the ratio of rates of substitution at loci with selection coefficient *s* to that at selectively neutral loci with the same mutation rate is 4*N*_e_*s*/[1 − exp(−4*N*_e_*s*)], assuming that 4*N*_e_*s* did not change for a long time. From this ratio, which can be obtained from the data presented in figure 2 or 3, we can recover numerically the value of 4*N*_e_*s* at each nucleotide position. Assuming that corresponding positions of all SSs experience approximately the same fitness landscape, we expect that the absolute values of 4*N*_e_*s* for positive selection acting against the Nc alleles must equal those for negative selection protecting the Cn alleles (Charlesworth and Eyre-Walker 2007).

**Fig. 4.—:**
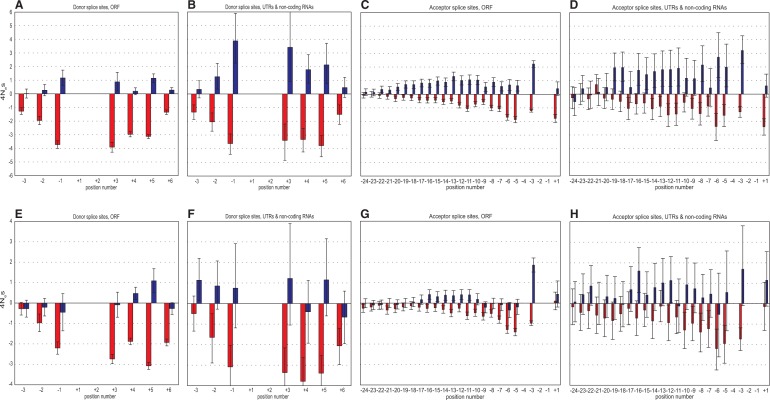
Strength of selection. Strength of selection measured in 4*N*_e_*s* units (vertical axis) acting on Nc-to-Cn substitutions (blue bars), and Cn-to-Nc substitutions (red bars). Positive and negative values of 4*N*_e_*s* correspond to positive and negative selection, respectively. (*A–D*) The *Homo sapiens* lineage; (*E–H*) the *Drosophila melanogaster* lineage. Columns are as in figure 2. Error bars are 95% confidence intervals.

The data show that donor and acceptor SSs, as well as coding and noncoding SSs, are under different modes of selection (fig. 4). Acceptor SSs are generally characterized by similar strengths of negative selection protecting the Cn alleles, and of positive selection acting against the Nc alleles. This is in agreement with what is expected under constant weak selection that is homogeneous across all acceptor SSs. Nevertheless, at a substantial fraction of positions, the strength of negative selection in favor of Cn exceeds the strength of positive selection than would lead to its establishment. This is true for positions −6, −5, and +1 of the acceptor SSs. It is also true for most positions of the donor SSs, especially in the coding SS in the human lineage, and in the noncoding SSs in the *D. melanogaster* lineage.

Thus, in many of the positions, Nc nucleotides are more conserved than neutrally expected. This fact clearly indicates that selection does not always favor the maximal strength of an SS, either because the strong SSs are deleterious by themselves (e.g., because excess site strength impedes regulation; Zheng et al. 2000; Ast 2004; Garg and Green 2007) or because of other competing selective constraints (Kotelnikova et al. 2005; Stergachis et al. 2013). Within exonic parts of SSs, such constraints may arise from negative selection on codon usage; in both intronic and exonic parts of SSs, they may also arise due to selection on splicing regulators or some other noncoding function (e.g., Stergachis et al. 2013). Under this scenario, an SS position cannot be properly described by a single universal Cn; instead, slightly different sequences may be favored within individual SSs.

### Site-Specific Negative Selection Favoring Nc Nucleotides

To better understand the role of negative selection, we compared the multispecies conservation of sets of nucleotides with that of the same nucleotides within nearly neutrally evolving regions. We analyze the conservation of a nucleotide that is present in *H. sapiens* or *D. melanogaster* by comparing it with 45 other vertebrate species or 15 other insect species, respectively, at a range of phylogenetic distances (see Materials and Methods for details). We considered the conservation *c*_nuc_ of the specific Cn (Nc) nucleotide present in *H. sapiens* or *D. melanogaster* at a given SS position, relative to that expected neutrally; and also the conservation *c*_set_ of all nucleotides defined as Cn (Nc) for this position.

As expected, the Cn nucleotides observed in SSs of *H. sapiens* or *D. melanogaster* are always more conserved than in neutrally evolving sequences (supplementary table S1, Supplementary Material online). Moreover, in most positions with two Cn nucleotides (see fig. 1), *c*_set_ defined for both these Cn nucleotides was higher than the *c*_nuc_ defined for the specific Cn nucleotide present, showing that selection favors any Cn nucleotide without distinguishing between them.

**Fig. 5.—:**
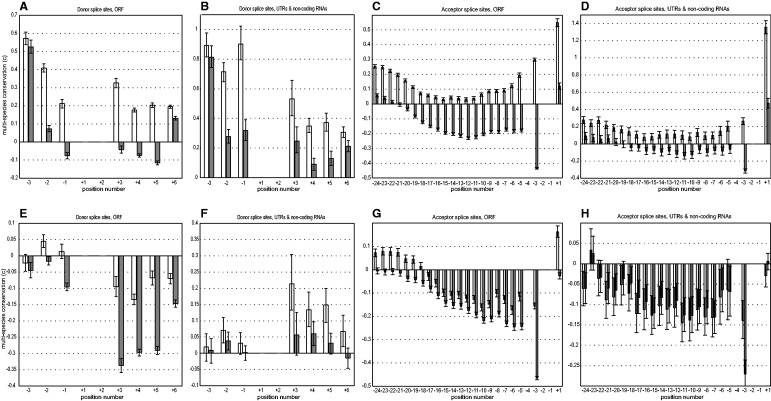
Multispecies conservation of Nc nucleotides. The values of *c = *(*obs* − *exp*)/*exp* of Nc nucleotides are shown, where *obs* is the observed mean multispecies conservation, and *exp* is the expected mean multispecies conservation (neutral control). Conservation was calculated for the specific Nc nucleotide present in *Homo sapiens* or *Drosophila melanogaster* (*c*_nuc_, white bars) or for all nucleotides defined as Nc for this position (*c*_set_, grey bars). (*A–D*) *Homo sapiens*; (*E–H*) *D. melanogaster*. (*A*), (*B*), (*E*), (*F*): Donor SSs; (*C*), (*D*), (*G*), (*H*): Acceptor SSs. (*A*), (*C*), (*E*), (*G*): Coding SSs; (*B*), (*D*), (*F*), (*H*): Noncoding SSs. Error bars are 95% confidence intervals generated using bootstrapping.

The pattern observed for the Nc nucleotides is more complicated. Within the positions of SS in *H. sapiens* currently occupied by the Nc nucleotide*,* this nucleotide is more conserved than neutrally expected *(c*_nuc_ > 0; fig. 5*A* and *D*; supplementary table S1, Supplementary Material online). Above-neutral conservation implies that, in some of the SSs, the currently existing Nc nucleotides are protected by negative selection. Often, the same holds for the set of Nc nucleotides as a whole (*c*_set_ > 0); therefore, even the Nc→Cn substitutions are selected against. Still, this negative selection frequently favors the specific Nc nucleotide observed in *H. sapiens,* rather than any Nc nucleotide, as indicated by the fact that *c*_nuc_ > *c*_set_ in all human SSs.

Therefore, the individual positions of donor SSs are under conflicting selection pressures: In most of them, the Cn nucleotide is favored, whereas in some, the Nc nucleotide is favored. The SSs in which the Nc nucleotide is observed in *H. sapiens* are enriched in those where Nc is beneficial, leading to their above-neutral overall conservation (fig. 5*A*–*D*). This violates our initial assumption that corresponding positions within different SSs experience the same fitness landscape throughout the genome. Pooling together SSs from these two classes causes the mismatch between the observed strength of negative selection maintaining the current Cn nucleotide, and of positive selection leading to its establishment (fig. 4*A*, *B*, *E*, and *F*).

In *D. melanogater,* both *c*_nuc_ and *c*_set_ for Nc nucleotides can be either positive or negative (fig. 5*E*–*H*), indicating that the universal selection pressure favoring the Cn nucleotide can override any conflicting site-specific selection favoring the Nc nucleotide. Still, the specific Nc nucleotide observed at a given SS is usually more conserved than the set of all Nc nucleotides *(c*_nuc_ > *c*_set_), indicating that some site-specific selection may be present. The difference between the SSs in *H. sapiens* and *D. melanogater* implies that such site-specific selective constraint is more prevalent in the former than in the latter.

There are two (nonmutually exclusive) explanations for the fact that some Nc nucleotides evolve under negative selection. First, strong SSs (i.e., those with a high number of Cn nucleotides) are not always the most fit; in some cases, weak SSs are selected for. For instance, cassette-exon SSs seem to be under selection to be weak (Garg and Green 2007), and several experimental studies showed that mutations in weak alternative SSs that make them closer to the consensus may cause loss of splicing regulation (Zheng et al. 2000; Ast 2004). Second, the current Nc nucleotide may be preferred because of other competing selective constraints, possibly not directly connected to splicing itself. The fact that the selection favoring the particular existing Nc nucleotide exceeds the selection favoring the Nc nucleotides per se suggests that the nucleotide identity, rather than its belonging to the Nc class, is what primarily determines its conservation; and thus argues for the second explanation, and against the first one. The negative correlation between the SS strength and the conservation of Cn nucleotides (see below) also argues against the first explanation.

### Other Differences between SSs

Both in human and in *D. melanogaster*, the observed patterns were similar between the regions of high and low recombination (supplementary fig. S1, Supplementary Material online), which means that they are not due to selection acting at linked sites. Furthermore, in mammals, these patterns do not change when CpG dinucleotides are excluded, so CpG hypermutability plays no role here; supplementary fig. S2, Supplementary Material online)

**Fig. 6.—:**
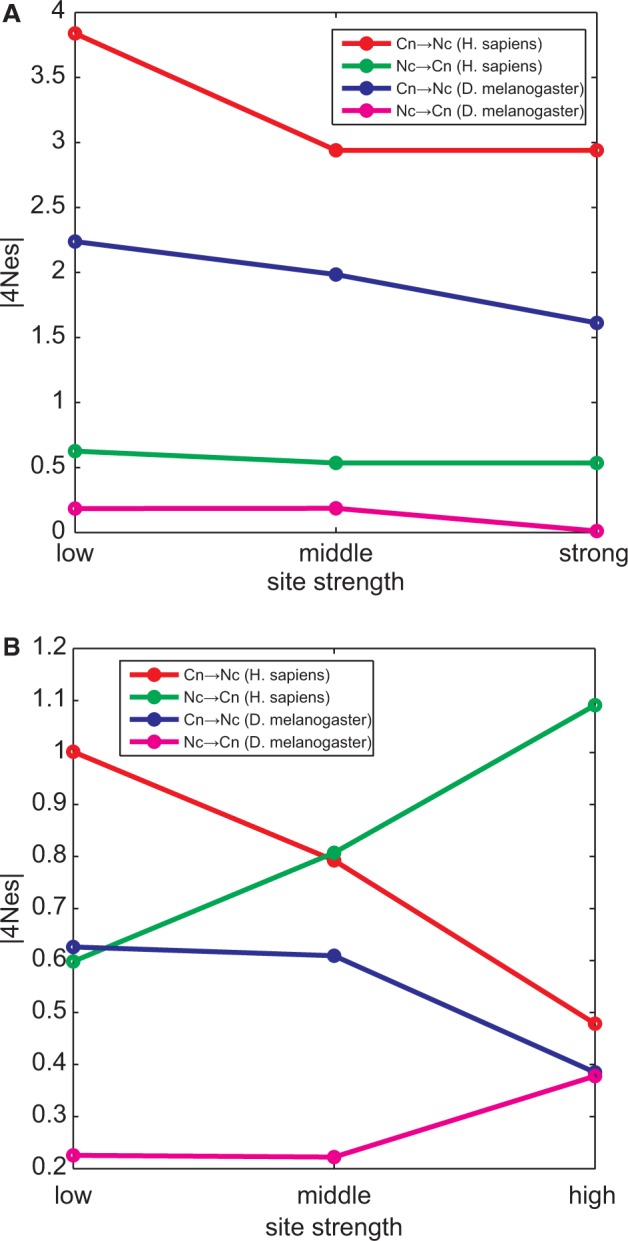
Dependence of efficiency of selection on SS strength. Average efficiency of selection acting on Cn-to-Nc substitutions (red and blue lines) and Nc-to-Cn substitutions (green and magenta lines) in the *Homo sapiens* lineage (red and green lines) and the *Drosophila melanogaster* lineage (blue and magenta lines), expressed as absolute values of 4*N*_e_*s* averaged through all SS positions. All SSs was subdivided into three classes (low, middle, and high) depending on SS strength (i.e., the fraction of the Cn nucleotides). (*A*) Donor SSs; (*B*) acceptor SSs.

We compared selection acting on SSs varying the SS strength (the current fraction of the Cn nucleotides) and the expression level of the gene, and also compared our sample of constitutively spiced exons with the alternatively spliced ones. We did not find significant systematic differences between strong, intermediate and weak SSs (fig. 6), between SSs of constitutive and cassette exons (supplementary fig. S3, Supplementary Material online), or between SSs from genes with high, middle or low expression levels (supplementary fig. S4, Supplementary Material online). The only exception was the observed dependence of negative selection for Cn on SS strength, with the Cn nucleotides favored to a higher degree at weak SSs (fig. 6). Finally, the results were approximately independent on whether maximum likelihood or maximum parsimony was used for reconstruction of substitutions (supplementary fig. S5, Supplementary Material online). In some of the comparisons, slightly stronger evidence for positive selection, and/or weaker evidence for negative selection, was observed under maximum likelihood reconstruction; this is probably due to maximum parsimony underestimating the number of Nc→Cn substitutions, leading to slight overestimation of negative selection, and underestimation of positive selection.

Recently, Irimia et al. (2009) analyzed the Nc→Cn and Cn→Nc substitutions in the SSs. Similar to our results, they observed negative selection favoring the Cn nucleotides; however, in their analysis, the rates of the Nc→Cn substitutions were indistinguishable from the neutral rates. There are a number of differences between our analysis and that of Irimia et al. First, they only analyzed the intronic parts of donor SSs. We took into account intronic as well as exonic part of both donor and acceptor SSs (fig. 4). Second, we used only constitutive donor and acceptor SSs, whereas Irimia et al. did not distinguish between splicing types. Although constitutive and alternative SSs showed no significant difference in our comparison (supplementary fig. S3, Supplementary Material online), a fraction of the alternative SSs may be under substantial selection for splicing regulation, possibly reducing the signal of positive selection. We also treated separately the “noncoding” SSs from noncoding RNA segments (UTRs of mRNAs and noncoding RNAs) and the “coding” SSs within ORF-interrupting introns. Finally, we used a much larger data set of SSs from the GENCODE project.

### Genome-Wide Level of Selection at SSs

**Fig. 7.—:**
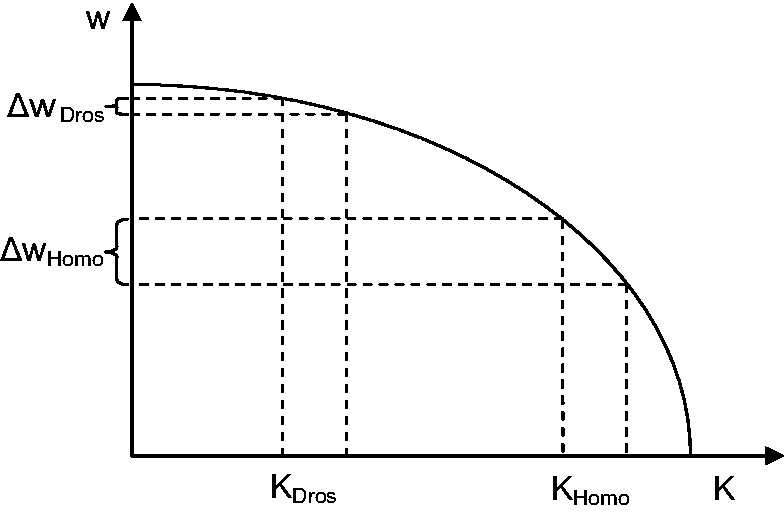
A hypothetical curve relating the fitness *w* to the number of Nc nucleotides *K* at an SS under synergistic epistasis. A single Nc-to-Cn mutation in *Homo* leads to a more drastic loss in fitness than the same mutation in *Drosophila*.

Both in *Homo* and in *Drosophila,* positive and negative selection is mostly of the strength 1 < |4*N*_e_*s*| < 4 (fig. 4). However, *N*_e_ is only approximately 1 × 10^4^ in *Homo,* compared with approximately 1 × 10^6^ in *Drosophila* (Charlesworth 2009). The rate of fixation of weakly selected mutations may be decoupled from *N*_e_ by genetic draft (Gillespie 2001); however, draft should also depend on the local recombination rate, and we see no difference between regions of high and low recombination in our patterns (supplementary fig. S1, Supplementary Material online). Therefore, the observed values imply that selection favoring the Cn nucleotide within SSs is at least approximately 2 × 10^−5^ in humans, but only approximately 2 × 10^−7^ in *Drosophila*. One reason for this difference may be the Li–Akashi effect: A functional unit accumulates deleterious replacements until, under synergistic epistatis, the effect of a replacement on fitness becomes large enough for selection to successfully oppose it, which happens when *N*_e_*s* becomes of the order of 1 (Li 1987; Akashi 1995; Kondrashov 1995; Goldstein 2013); that is, at substantially lower strengths of selection in *Drosophila* than in human (fig. 7). If the function that relates mutation-associated selection with the current fitness is the same in the two species, for the Li–Akashi effect to be in place, we expect the SSs in *Homo* to be, on average, weaker than those in *Drosophila*; within each species, we also expect weaker SSs to be more intolerant to further addition of mutations that reduce their fitness (fig. 7)*.* Consistently, the fraction of nucleotide positions of acceptor SSs occupies by Cn nucleotides is slightly lower in the *Homo* genome (77.8%) than in the *Drosophila* genome (90.1%), although the same does not hold for the donor SSs (78.7% vs. 78.6%). Furthermore, the strength of negative selection acting against Cn→Nc substitutions tends to be negatively correlated with the SS strength (fig. 6). Still, the differences between the SSs in *Homo* and in *Drosophila* appear to be too weak to explain the 100-fold disparity in selection acting against mutations in them, and this disparity remains somewhat of a mystery.

The deleterious Nc nucleotides that occupy a substantial fraction of positions within SSs imply a considerable genetic load. In total, human genome carries approximately 150,000 of constitutive donor SSs, and approximately 150,000 of constitutive acceptor SSs. An average donor SS carries 2.92 Nc nucleotides, and an average acceptor SS, 7.10 Nc nucleotides. As the per locus selection coefficient is approximately 2 × 10^−5^, the sum of selection coefficients against all Nc alleles at these loci are approximately 10 and approximately 20. These values may be somewhat overestimated due to the instances of Nc nucleotides that are not selected against; still, this load is too high, unless it is alleviated by selection being globally epistatic rather than multiplicative, and/or stabilizing rather than purifying (Kondrashov 1995; Charlesworth 2013). Analogous estimation for *D. melanogaster* gives the load approximately 2 × 10^−2^ and approximately 7 × 10^−2^ for constitutive donor and acceptor SSs, respectively.

In summary, we have shown that the nucleotides in the SSs that determine the splicing efficiency experience weak selection. Although Nc nucleotides are favored in some of the individual SSs, overall, they are disfavored, and Cn nucleotides are favored, by selection. The weakness of this selection results in a steady-state turnover of very slightly deleterious and beneficial substitutions. Under the resulting equilibrium, a high fraction of SSs is occupied by suboptimal nucleotides, leading to a substantial genome-wide genetic load.

## Supplementary Material

Supplementary tables S1 and S2 and figures S1–S5 are available at *Genome Biology and Evolution* online (http://www.gbe.oxfordjournals.org/).

Supplementary Data
