# Supplementary material for: Weak Negative and Positive Selection and the Drift Load at Splice Sites
Source: Genome Biol Evol. 2014 May 14;6(6):1437–47. doi: 10.1093/gbe/evu100 (PMC4079205; doi:10.1093/gbe/evu100)
Supplement: Supplementary Data [file supp_6_6_1437__index.html]

Weak negative and positive selection and the drift load at splice sites — Weak Negative and Positive Selection and the Drift Load at Splice Sites — Supplementary Data 

# Weak Negative and Positive Selection and the Drift Load at Splice Sites

## Supplementary Data

files

**Files in this Data Supplement:**

- Supplementary Data - zip file
